# Bioelectrical impedance analysis and physical function improved risk prediction for cardiac dysfunction in hemodialysis patients

**DOI:** 10.3389/fphys.2026.1786252

**Published:** 2026-03-19

**Authors:** Weichen Zhang, Lu Yao, Qin Zhu, Jianxin Lu, Qianhong Liu, Wei Ding

**Affiliations:** 1 Division of Nephrology, Shanghai Ninth People’s Hospital, School of Medicine, Shanghai Jiaotong University, Shanghai, China; 2 Division of Nephrology, Shanghai Zhongye Hospital, Shanghai, China

**Keywords:** bioelectrical impedance analysis (BIA), physical function, cardiac function, echocardiography, hemodialysis

## Abstract

**Introduction:**

Cardiovascular disease is the leading cause of death in maintenance hemodialysis (MHD) patients. This cross-sectional study investigated the associations between bioelectrical impedance analysis (BIA) parameters, physical function, and cardiac function in MHD patients, with the aim of identifying predictors of cardiac dysfunction.

**Methods:**

The study included 130 MHD patients from Shanghai Ninth People’s Hospital on December 2022. BIA measurements were executed using the Body Composition Monitor. Physical function was assessed by handgrip strength and natural gait speed. Cardiac systolic and diastolic functions were evaluated by echocardiography. Univariate and multivariable logistic regression analyses were performed to identify determinants of cardiac dysfunction.

**Results:**

Mean age of all participants was 62.39 ± 13.91 years old while 78 (60%) were male. Univariate and multivariable logistic regression analyses were performed to identify determinants of cardiac dysfunction. Multivariable analysis revealed that NT-proBNP (odds ratio [OR], 1.098; 95% CI, 1.011-1.193; *P* = 0.027) and natural gait speed (OR, 0.002; 95% CI, 0.001-0.528; *P* = 0.033) were independent determinants of impaired systolic function. Their combination had a high predictive value (area under the curve [AUC] = 0.854; *P* < 0.001). Total body water (OR, 1.104; 95% CI, 1.010-1.206; *P* = 0.029) and NT-proBNP (OR, 1.078; 95% CI, 1.010-1.151; *P* = 0.025) were significant determinants of impaired diastolic function, with the combined prediction (AUC = 0.746; *P* < 0.001) outperforming either marker alone.

**Discussion:**

BIA and physical function parameters improved risk stratification beyond conventional biomarkers, providing practical tools for screening cardiac risk in MHD patients.

## Introduction

Cardiovascular disease remains the leading cause of mortality in patients with end-stage renal disease (ESRD) undergoing maintenance hemodialysis (MHD), with cardiac dysfunction representing a critical pathological manifestation (Harris et al., 2019). Both systolic and diastolic impairments independently predict adverse outcomes in this population, yet routine echocardiographic monitoring in clinical practice is limited by operational complexity and potential measurement variability (Chiu et al., 2014; Domjanović Matetić et al., 2024).

Bioelectrical impedance analysis (BIA) is a noninvasive, bedside method for assessing fluid status and body composition. These parameters may reflect cardiovascular stress. For example, fluid overload has been shown to accelerate ventricular remodeling, while malnutrition–inflammation complex syndrome contributes to myocardial wasting (Tsilonis et al., 2016). BIA is widely used to guide the management of hemodialysis patients. Parameters such as extracellular-to-total body water ratio and overhydration values help identify fluid overload, a common complication in this population (Yılmaz et al., 2014; Lv et al., 2025). Therefore, clinicians can adjust ultrafiltration volume based on BIA-guided dry weight assessment. A meta-analysis of randomized controlled trials (RCTs) demonstrated that BIA-guided dry weight management reduced cardiovascular events by 21% and mortality by 33% (Wathanavasin et al., 2025). In addition, BIA contributes to nutrition assessment. For example, phase angle may be associated with protein intake and muscle loss (Ding et al., 2022), while the body composition monitor (BCM) reflects metabolically active tissue and correlates with nutritional status (Oliveira et al., 2020).

Physical function is also a critical measure in hemodialysis patients. Handgrip strength (HGS) reflects overall muscle strength, and reduced HGS is associated with protein–energy wasting and malnutrition–inflammation complex syndrome (Chavez-Mendoza et al., 2022; Dilloway et al., 2023). Gait speed serves as an integrative indicator of cardiopulmonary health in patients with chronic diseases such as cancer and chronic obstructive pulmonary disease (Walsh et al., 2021; Ohno et al., 2023). Both HGS and gait speed are widely used in clinical and research settings for risk stratification and for monitoring interventions in hemodialysis patients.

Evidence also links BIA and physical function with cardiovascular adverse outcomes. An observational study of 161 adult hemodialysis patients found that BIA-derived phase angle predicted cardiovascular events (Wang et al., 2024), an association corroborated by other studies (Paglialonga et al., 2012; Shin et al., 2017). Physical function has also been linked to cardiovascular outcomes. For example, a prospective cohort study by Kim et al. (2019) showed that low HGS was independently associated with cardiovascular events, while a clinical trial by Kuki et al. (2019) demonstrated that reduced gait speed and decreased HGS were independent predictors of both fatal and nonfatal cardiovascular events in hemodialysis patients.

Although associations between conventional biomarkers and cardiac function have been established, the predictive utility of combining BIA-derived parameters with physical function assessments in MHD patients remains underexplored. Therefore, this study aimed to investigate these relationships and identify accessible clinical tools for risk stratification.

## Materials and methods

This single-center, cross-sectional study was conducted at Shanghai Ninth People’s Hospital, affiliated with Shanghai Jiao Tong University School of Medicine. The Ethics Committee on Human Research at Shanghai Ninth People’s Hospital approved the study (SH9H-2020-T439-3). All participants provided written informed consent.

### Study population

Hospital records of hemodialysis patients on December 2022 were reviewed. Patients older than 18 years who had been receiving MHD for more than three months were included. Exclusion criteria were pregnancy, a diagnosis of malignant tumor, a history of joint replacement, hemiplegia, or incomplete data for the BIA test or physical performance assessment. After applying these criteria, 130 patients were included in the study.

### Clinical data collection

Demographic and biochemical data were obtained from the hospital medical record system. Demographic data included age, gender, height, body weight, and primary disease. Body mass index (BMI) was calculated as weight (kg) divided by height squared (m^2^). The most recent hematology test results were recorded, including hemoglobin, serum albumin, blood urea nitrogen (BUN), serum creatinine, uric acid, creatine kinase-MB (CK-MB), myoglobin, and N-terminal pro-brain natriuretic peptide (NT-proBNP). Hemodialysis data included dialysis access, dry weight, and pre- and post-dialysis BUN.

### Bioelectrical impedance analysis and physical function tests

BIA was performed using the Body Composition Monitor (Fresenius Medical Care AG, Bad Homburg v.d. Höhe, Germany), a multifrequency bioimpedance spectroscopy device. Measurements were taken in the right calf at four frequencies (5, 50, 100, and 200 kHz) after hemodialysis. Extracellular water (ECW), intracellular water (ICW), and total body water (TBW) were measured. Lean tissue mass (LTM) and fat tissue mass (FTM) were also assessed.

Physical function was evaluated using HGS and gait speed. HGS was measured in the arm without an arteriovenous fistula (AVF) before the hemodialysis session using a digital hand dynamometer (Jamar Plus, Performance Health, Illinois, USA). Gait speed was assessed on the treatment day before hemodialysis by measuring walking speed over a 4-meter course at the participant’s usual pace followed standardized protocol without walking aids or escort (Cruz-Jentoft et al., 2010; Chen et al., 2014). The test was repeated three times, and the average speed was used for analysis.

#### Echocardiography

All participants underwent transthoracic echocardiography within six months before December 2022. Left ventricular systolic, diastolic functions and cardiac valve calcification were assessed by Philips EPIQ CVx (Philips Medical Systems, Andover, MA, USA). Recorded parameters included ejection fraction (EF), interventricular septal thickness, posterior wall thickness, left ventricular end-diastolic diameter (LVEDD), peak early (E) and late (A) mitral diastolic velocities and their ratio (E/A), and the ratio of peak early mitral diastolic velocity to early tissue-Doppler mitral diastolic velocity (E/e’). Impaired systolic function was defined as EF < 50%, and impaired diastolic function was defined as E/A > 2 or E/e’ > 12 (Nagueh et al., 2016; Loutradis et al., 2018).

### Statistical analysis

Standard single-pool Kt/V (spKt/V) was calculated as –ln [(R − 0.008 × t)/(1 – 0.025 × R)], where R is the ratio of post-dialysis to pre-dialysis BUN, and t is the dialysis session duration (hours) (Daugirdas, 1993). Left ventricular mass (LVM) was calculated as 0.8 × [1.04 × ((IVSd + LVIDd + LVPWd)^3^ − LVIDd^3^)] + 0.6, where IVSd is interventricular septal thickness in diastole, LVIDd is left ventricular internal diameter in diastole, and LVPWd is left ventricular posterior wall thickness in diastole. All these diastole parameters were measured during echocardiography (Lang et al., 2015).

Data were analyzed using SPSS for Windows, Version 15.0 (SPSS Inc., Chicago, IL, USA). Value of NT-proBNP is reduced by 1000 times and displayed by the unit “per 1000 pg/mL” in order to show OR normally. Continuous variables are presented as means ± standard deviation or medians with interquartile ranges, depending on distribution. Categorical variables are presented as frequencies and percentages. Univariate logistic regression was used to assess demographic and clinical variables as candidate predictors of cardiac systolic and diastolic dysfunction. Variables with *P* < 0.1 were included in the multivariable regression model. Backward elimination was applied to reach the final model. Receiver operating characteristic (ROC) curves were generated to determine the predictive value for clinical application.

## Results

### Patient demographics

The demographic and clinical characteristics of the study participants (*n* = 130) are presented in **Table 1**. The mean age was 62.39 ± 13.91 years, and 78(60%) patients were male. The mean BMI was 23.33 ± 4.63 kg/m^2^. The most common primary disease was hypertensive nephropathy (41, 31.5%), followed by primary glomerulonephritis (29, 22.3%). A total of 111 patients (85.4%) used AVF for hemodialysis, and the mean Kt/V was 1.68 ± 0.81.

**Table 1:** Demographic and clinical characteristics of hemodialysis patients (N = 130).

Characteristics	Mean ± SD/n (%)
Age (years)	62.39 ± 13.91
Male gender	78 (60%)
BMI (kg/m2)	23.33 ± 4.63
Primary disease	
*Hypertensive nephropathy*	41 (31.5%)
*Glomerulonephritis*	29 (22.3%)
*Diabetic nephropathy*	28 (21.5%)
*Polycystic kidney*	9 (6.9%)
*Others*	23 (17.7%)
Comorbidities	
*Hypertension*	86 (66.2%)
*Diabetes mellitus*	36 (27.7%)
*Coronary heart disease*	32 (24.6%)
Kt/V	1.68 ± 0.81
Laboratory parameters	
Hemoglobin (g/L)	102.66 ± 16.77
Serum albumin (g/L)	37.72 ± 3.69
BUN (mmol/L)	24.84 ± 7.33
SCr (umol/L)	959.30 ± 285.26
UA (umol/L)	449.82 ± 90.05
CK-MB (U/L)	1.97 ± 1.33
Myoglobin (g/L)	155.15 ± 64.51
NT-proBNP(pg/ml)	4855.5(2429.0,12341.5)
BIA parameters	
TBW (L)	31.00 ± 6.30
ECW (L)	14.31 ± 3.31
ICW (L)	16.70 ± 3.58
LTM (kg)	33.67 ± 8.79
FTM (kg)	21.91 ± 9.23
Echocardiographic parameters	
LVM (g)	184.62 ± 62.75
LVEDD (mm)	48.73 ± 6.51
EF (%)	58.70 ± 7.92

BMI, body mass index; Kt/V, urea clearance index representing dialysis dose (K, urea clearance; t, dialysis time; V, volume of urea distribution); BUN, blood urea nitrogen; SCr, serum creatinine; UA, uric acid; CK-MB, creatine kinase-MB; NT-proBNP, N-terminal pro-brain natriuretic peptide; BIA, bioelectrical impedance analysis; TBW, total body water; ECW, extracellular water; ICW, intracellular water; LTM, lean tissue mass; FTM, fat tissue mass; LVM, left ventricular mass; LVEDD, left ventricular end-diastolic diameter; EF, ejection fraction.

### BIA, echocardiographic, and physical function parameters

Laboratory test results are shown in **Table 1**. Mean values included hemoglobin (102.66 ± 16.77 g/L), serum albumin (37.72 ± 3.69 g/L), CK-MB (1.97 ± 1.33 U/L), and myoglobin (155.15 ± 64.51 g/L). The median NT-proBNP level was 4855.5 pg/mL (interquartile range, 2429.0-12341.5 pg/mL).

BIA parameters (**Table 1**) included mean TBW (31.00 ± 6.30 L), ECW (14.31 ± 3.31 L), and ICW (16.70 ± 3.58 L). The LTM was 33.67 ± 8.79 kg, and the FTM was 21.91 ± 9.23 kg.

Echocardiographic findings included a mean EF of 58.70 ± 7.92%, LVM of 184.62 ± 62.75 g, and LVEDD of 48.73 ± 6.51 mm. Impaired systolic function was diagnosed in 17% of patients, and impaired diastolic function was diagnosed in 30%. Cardiac valve calcification was present in 28%.

Physical function assessment showed a mean HGS of 27.63 ± 8.19 kg, and a mean gait speed of 0.94 ± 0.20 m/s.

### Association of NT-proBNP, and gait speed with impaired cardiac systolic function

Univariate and multivariable logistic regression results for impaired cardiac systolic function are presented in **Table 2**. Univariate analysis showed that male gender, NT-proBNP, TBW, ECW, LTM, and gait speed were associated with impaired systolic function. Multivariable logistic regression analysis indicated that NT-proBNP (odds ratio [OR], 1.098; 95% CI, 1.011-1.193; *P* = 0.027) and gait speed (OR, 0.002; 95% CI, 0.001–0.528; *P* = 0.033) were independent determinants of impaired systolic function.

**Table 2:** Univariable and multivariable logistic regression models of cardiac systolic function.

Characteristics	Univariable			Multivariable		
	OR	P	95% CI	OR	P	95% CI
Age	0.988	0.537	0.950-1.027			
Male gender	5.25	0.036	1.116-24.706			
Kt/V	0.532	0.228	0.191-1.484			
Hemoglobin	0.983	0.387	0.947-1.021			
Serum albumin	0.871	0.093	0.741-1.024			
CK-MB	1.138	0.577	0.723-1.789			
Myoglobin	1.006	0.139	0.998-1.013			
NT-proBNP, per 1000pg/ml	1.115	<0.001	1.057-1.176	1.119	0.002	1.044-1.200
BMI	1.06	0.341	0.940-1.196			
TBW	1.135	0.015	1.025-1.258			
ECW	1.217	0.035	1.014-1.461			
OH	1.351	0.149	0.898-2.033			
OH/ECW	1.042	0.17	0.982-1.105			
LTM	1.094	0.019	1.015-1.178			
FTM	0.992	0.793	0.932-1.055			
HGS	1.05	0.221	0.971-1.134			
Gait speed	0.003	<0.001	0.001-0.172	0.003	0.025	0.001-0.472

BMI, body mass index; Kt/V, urea clearance index representing dialysis dose (K, urea clearance; t, dialysis time; V, volume of distribution of urea); CK-MB, creatine kinase-MB; NT-proBNP, N-terminal pro-brain natriuretic peptide; TBW, total body water; ECW, extracellular water; OH, overhydration; LTM, lean tissue mass; FTM, fat tissue mass; HGS, handgrip strength.

**Figure 1:**
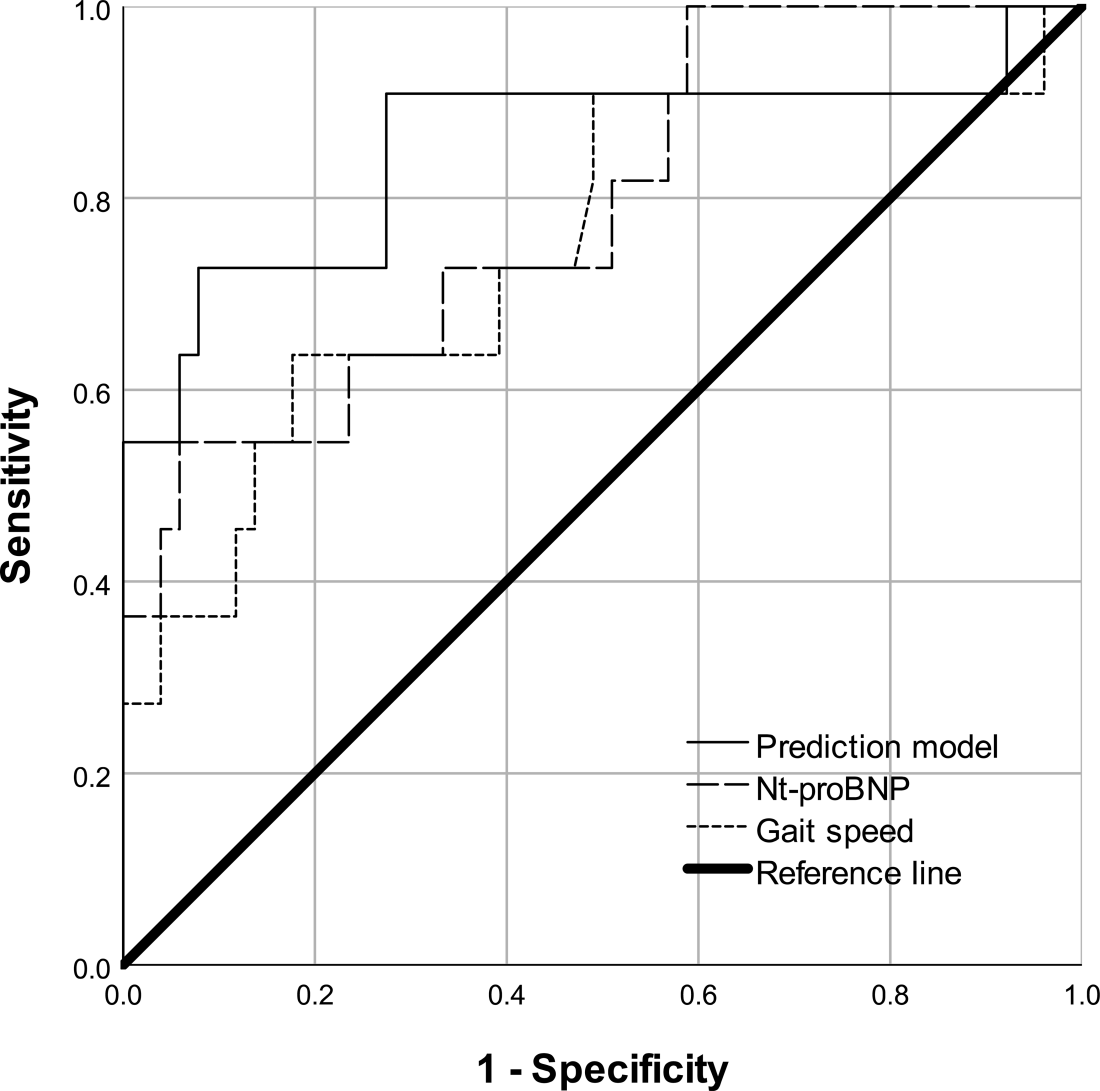
ROC curve of NT-proBNP, natural gait speed, and their combination for predicting impaired cardiac systolic function in MHD patients. ROC, receiver operating characteristic; NT-proBNP, N-terminal pro-brain natriuretic peptide; MHD, maintenance hemodialysis.

ROC curve analysis (**Figure 1**, **Table 3**) and discriminatory performance of NT-proBNP, gait speed, and their combination in predicting impaired cardiac systolic function showed that NT-proBNP (AUC, 0.783; 95% CI, 0.648-0.919; *P* = 0.001), and gait speed (AUC, 0.769; 95% CI, 0.604–0.934; *P* = 0.004) could predict impaired systolic function, with their combination performing best (AUC, 0.854; 95% CI, 0.693-0.994; *P* < 0.001).

**Table 3:** Discriminatory performance of NT-proBNP, gait speed and their combination in predicting MHD patients with impaired cardiac systolic function.

Predictor variables	AUC	P	95%CI
Multivariable prediction model	0.854	<0.001	0.693-0.994
NT-proBNP, per 1000pg/ml	0.783	0.001	0.648-0.919
Gait speed	0.769	0.004	0.604-0.934

The multivariable logistic prediction model included NT-proBNP and gait speed as independent variables.

AUC, area under the curve; NT-proBNP, N-terminal pro-brain natriuretic peptide; MHD, maintenance hemodialysis.

### Association of NT-proBNP and TBW with impaired cardiac diastolic function

Univariate and multivariable logistic regression results for impaired cardiac diastolic function are presented in **Table 4**. Univariate analysis showed that hemoglobin, NT-proBNP, BMI, TBW, ECW, OH, OH/ECW, and gait speed were associated with impaired diastolic function. Multivariable logistic regression analysis indicated that NT-proBNP (OR, 1.078; 95% CI, 1.010-1.151; *P* = 0.025) and TBW (OR, 1.104; 95% CI, 1.010-1.206; *P* = 0.029) were independent determinants of impaired diastolic function.

**Table 4:** Univariable and multivariable Logistic regression models of cardiac diastolic function.

Characteristics	Univariable			Multivariable		
	OR	P	95% CI	OR	P	95% CI
Age	1	0.997	0.971-1.030			
Male gender	1.339	0.493	0.581-3.087			
Kt/V	0.752	0.242	0.467-1.212			
Hemoglobin	0.961	0.009	0.933-0.990			
Serum albumin	0.921	0.203	0.811-1.045			
CK-MB	1.386	0.095	0.945-2.034			
Myoglobin	1.003	0.389	0.996-1.010			
NT-proBNP, per 1000pg/ml	1.078	0.01	1.018-1.142	1.078	0.025	1.010-1.151
BMI	1.122	0.04	1.005-1.251			
TBW	1.118	0.007	1.103-1.214	1.104	0.029	1.010-1.206
ECW	1.287	0.003	1.091-1.519			
OH	1.440	0.025	1.048-1.978			
OH/ECW	1.053	0.019	1.008-1.100			
LTM	1.041	0.133	0.988-1.097			
FTM	1.045	0.088	0.993-1.100			
HGS	1.009	0.764	0.954-1.066			
Gait speed	0.018	0.007	0.001-0.343			

BMI, body mass index; Kt/V, urea clearance index representing dialysis dose (K, urea clearance; t, dialysis time; V, volume of distribution of urea); CK-MB, creatine kinase-MB; NT-proBNP, N-terminal pro-brain natriuretic peptide; TBW, total body water; ECW, extracellular water; OH, overhydration; LTM, lean tissue mass; FTM, fat tissue mass; HGS, handgrip strength.

**Figure 2:**
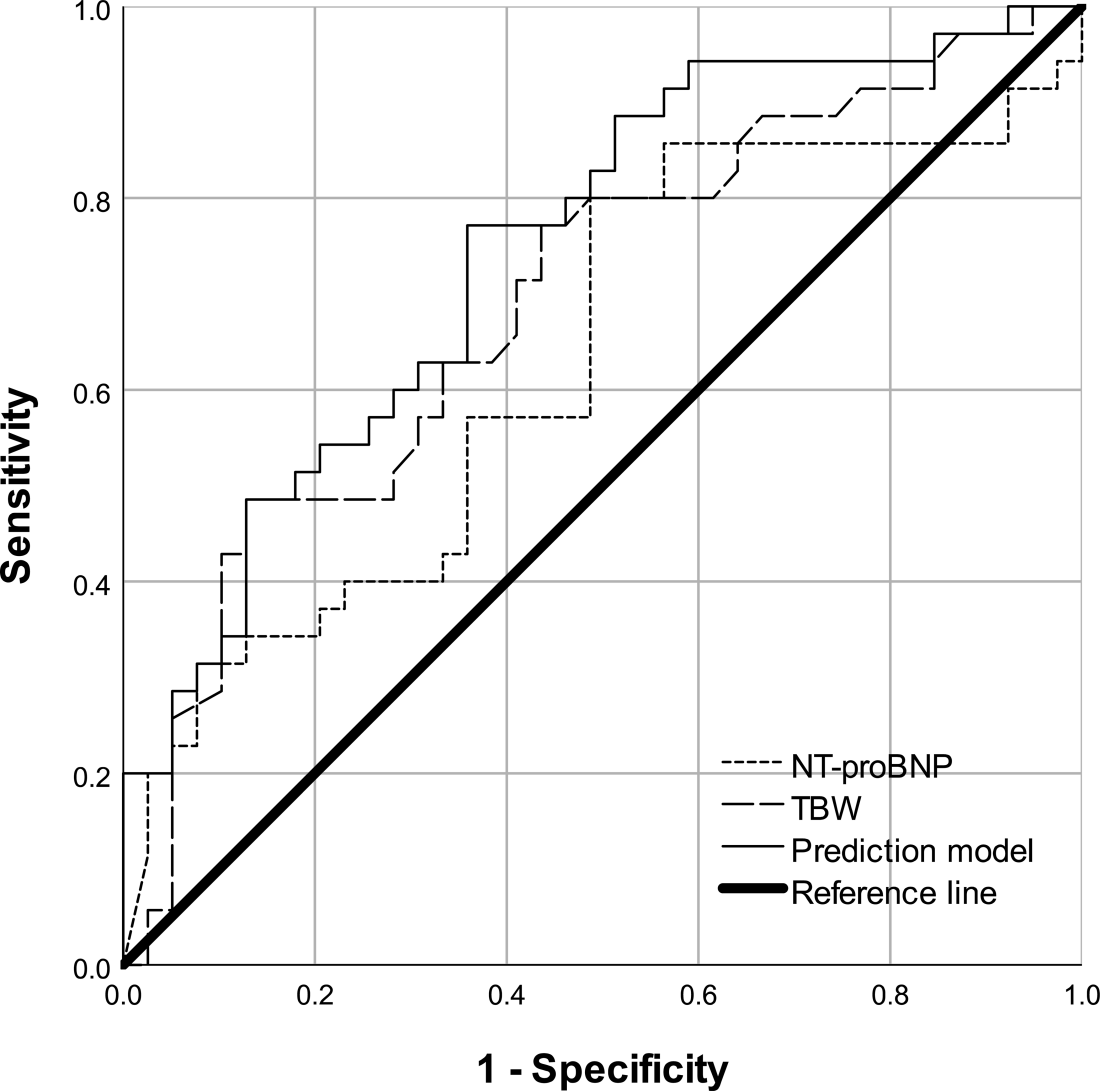
ROC curve of TBW, NT-proBNP, and their combination for predicting impaired cardiac diastolic function in MHD patients. ROC, receiver operating characteristic; NT-proBNP, N-terminal pro-brain natriuretic peptide; TBW, total body water; MHD maintenance hemodialysis.

ROC curve analysis (**Figure 2**, **Table 5**) showed that both NT-proBNP (AUC, 0.652; 95% CI, 0.536-0.767; *P* = 0.014) and TBW (AUC, 0.691; 95% CI, 0.575–0.807; *P* = 0.003) could predict impaired cardiac diastolic function, with their combination performing best (AUC, 0.746; 95% CI, 0.635-0.857; *P* < 0.001).

**Table 5:** Discriminatory performance of TBW, NT-proBNP and their combination in predicting MHD patients with impaired cardiac diastolic function.

Predictor variables	AUC	P	95% CI
Multivariable prediction model	0.746	<0.001	0.635-0.857
NT-proBNP, per 1000pg/ml	0.652	0.014	0.536-0.767
TBW	0.691	0.003	0.575-0.807

The multivariable logistic prediction model included NT-proBNP and TBW as independent variables.

AUC, area under the curve; NT-proBNP, N-terminal pro-brain natriuretic peptide; TBW, total body water; MHD, maintenance hemodialysis.

## Discussion

This cross-sectional study provides new insights on the relationships between BIA parameters, physical function, and cardiac dysfunction in MHD patients. Our key findings showed that: 1) NT-proBNP and gait speed were predictors of impaired cardiac systolic function; 2) TBW and NT-proBNP were associated with diastolic dysfunction; and 3) combined use of these accessible measures enhanced risk prediction beyond single predictor. These observations have important implications for practical risk assessment in resource-limited hemodialysis settings.

The strong association between gait speed and cardiac systolic function underscores its utility as an integrative functional biomarker. The predictive capacity of gait speed alone (AUC = 0.769) and in combination with other predictors (AUC = 0.854) suggests that physical function testing could effectively identify patients requiring advanced cardiac evaluation. Previous studies demonstrated that reduced gait speed predicted poor prognosis in patients with heart failure and those after cardiac surgery (Afilalo et al., 2018; Fuentes-Abolafio et al., 2020). Similar results were also observed in patients who underwent hemodialysis. Kutner et al. (2015) demonstrated that gait speed <0.6 m/s independently predicts markedly increased mortality (HR 2.17), framing walking as a systemic challenge that “challenges the heart, lungs, and circulatory systems.” Abe et al. (2016) directly established cardiac disease as an independent determinant of slow walking speed (OR 3.33), confirming that myocardial dysfunction constrains gait performance. However, these studies did not use echocardiography to evaluate cardiac function, partly because its implementation is complex. Our research showed that gait speed, an easily measured parameter, was associated with systolic dysfunction. This is particularly relevant considering the operational limitations of routine echocardiography in MHD patients.

We observed that both higher NT-proBNP levels and greater TBW were significantly associated with an increased odds of diastolic dysfunction. This appears to be similar as given established links between fluid overload and cardiac strain (Loutradis et al., 2020; Hanna et al., 2023; Faragli et al., 2025), which underscores the combined role of a well-established cardiac biomarker, reflective of ventricular wall stress, and a marker of overall body fluid status in the pathophysiology of this condition. While it is well-recognized that TBW expansion increases preload and left ventricular end-diastolic pressure, the precise sequence of myocardial remodeling in response to chronic volume excess remains incompletely characterized (Hanna et al., 2023). TBW elevation induces microvascular compression, subendocardial ischemia, and alterations in cardiomyocyte calcium handling—processes that may precede and potentiate the extracellular matrix fibrosis traditionally emphasized in heart failure with preserved ejection fraction (Loutradis et al., 2020). Physicians often simply use TBW to evaluate fluid overload or NT-proBNP to predict cardiac function in clinical practice. However, the ROC analysis in our research provided evidence for the predictive utility of these variables. While each marker alone demonstrated a significant but modest predictive ability, their combination yielded a substantially improved and statistically robust predictive performance. This suggests that assessing NT-proBNP and TBW concurrently offers superior discriminative power for identifying impaired cardiac diastolic function compared to using either marker in isolation. Furthermore, NT-proBNP was also proved to be related with cardiac systolic function, which highlighted its significance in the assessment of cardiac function.

Given that cardiovascular disease remains the leading cause of death in MHD patients, with substantially higher non-fatal cardiovascular event rates than in the general population (Pozzoni et al., 2004), early identification of declining cardiac function (Wang et al., 2024) is critical to improving prognosis. Unlike previous studies, this research focused on the decline of cardiac function in MHD patients rather than on cardiovascular events. We aimed to utilize simple monitoring methods such as BIA and physical function tests for early detection of impaired cardiac function—an aspect largely overlooked in prior research. Therefore, we established combined predictive models that offer several practical advantages. First, BIA and gait speed testing require minimal training compared to echocardiography. Second, equipment costs for BIA and gait speed testing are substantially lower than those for cardiac imaging devices and can easily be managed in resource-limited settings. Third, both tests can be performed during routine dialysis sessions, improving health monitoring efficiency.

Our study has several limitations. This cross-sectional study was based on clinical observational data, so various types of bias cannot be excluded. Single-center research also limits generalizability despite standardized protocols. To improve accuracy, our prediction models should be validated in larger populations. Finally, sample size constraints reduced the power for subgroup analyses, resulting in some confounders.

In conclusion, this study identified BIA-derived TBW and gait speed as potential predictors of cardiac dysfunction in MHD patients, with their combined application providing superior risk stratification. While echocardiography remains the gold standard for cardiac assessment, our findings support integrating BIA and functional testing as practical screening tools. This approach aligns with global initiatives to enhance ESRD care accessibility while addressing the operational challenges of specialized cardiac monitoring in this high-risk population.

## Data Availability

Publicly available datasets were analyzed in this study. This data can be found here: gump1015@163.com.
